# Parental Attitude towards the Provision of Nonsurgical Oral Health Care to Children with Oral Clefts: An Epidemiological Survey

**DOI:** 10.5005/jp-journals-10005-1051

**Published:** 2010-04-15

**Authors:** Manju Gopakumar, Amitha M Hegde

**Affiliations:** 1 Reader, Department of Pedodontics and Preventive Children Dentistry, AB Shetty Memorial Institute of Dental Sciences Mangalore, Karnataka, India; 2 Professor and Head, Department of Pedodontics and Preventive Children Dentistry, AB Shetty Memorial Institute of Dental Sciences, Mangalore, Karnataka, India

**Keywords:** Cleft lip/palate, dental treatment, parental attitude.

## Abstract

**Aim:**

To evaluate the attitudes of parents of 3 to 8 years old children with cleft lip and palate towards the provision of pediatric dental health care and assess the experience of dentistry in general dental practice.

**Materials and methods:**

A questionnaire was distributed to parents of 100 children in the age group of 3 to 8 years old with cleft lip and palate who visited the cleft lip and palate center in KS Hegde Medical Academy, Mangalore.

**Results:**

Out of the hundred patients, 66% of the patients registered directly at the cleft center for their deformity. Only 20% of these children visited a local dental practitioner for their dental health care. 42% had neither undergone any dental treatment nor received any dietary or oral hygiene advices. Regarding the provision of the dental treatment, 74% wanted a pediatric dentist to provide dental check-up and treatment at the cleft center, whereas, 24% preferred a dental practitioner close to their home and 2% does not want treatment anywhere.

**Conclusion:**

The survey indicates that there is parental support for the pediatric dental assessment at the cleft center with subsequent arrangement of dental treatment with their local dental practitioners. The majority wanted a pediatric dentist to provide the dental check-up and treatment at the cleft center.

## INTRODUCTION

Good and healthy children of today, make the better, strong and healthy parents of tomorrow. But, the environment within which the children are being raised is changing due to various reasons like single parent families, working mothers with diverse arrangements for childcare, continued poverty and increased incidence of child abuse and neglect. In such a world, the major brunt of responsibility to help these neglected children falls on the doctor’s shoulders. The condition of these children worsens when they are born with anomalies like the orofacial clefts. No congenital anomaly has more complexity and diverse morphological influence in the oral and maxillofacial region than the cleft of the lip and palate (CLP). It has an occurrence of 1 in 800 to 1000 live births and is the most common congenital facial anomaly.^1^ Studies have shown that children with CLP have poor dental health than children in the general population.^2-4^

Achieving optimal dental health may be difficult due to anatomy of the cleft area, malaligned teeth, hypoplastic defects and scarring.^2^ Children with CLP tend to have poorer oral and gingival health compared with those with isolated cleft of the lip or palate.^5^

The special needs of the children with CLP have been recognized by the Clinical Standards Advising Group (CSAG). Their report recommended that preventive advice should be given to the parents of newly born cleft children within one year and pediatric dental services provided throughout childhood and adolescence.^6^

As the incidence of cleft lip is only 1/800 live births, a general dental practitioner may have limited experience in treating these patients^7^ and as a result may encounter more management problems.^8^ As a member of the *cleft team,* a pediatric dentist can provide preventive programs and dietary advice to cleft lip and palate children which have shown to reduce dental caries^9^ and thus be cost effective.^10^

The aim of the study was to evaluate the attitudes of the parents of the children with CLP towards the provision of pediatric dental care and to assess their experience of dentistry in general dental practice.

## SUBJECTS AND METHODS

A structured questionnaire was given to the parents of hundred children who visited the cleft lip center in KS Hegde Medical Academy, Mangalore. The children were in the age group of 3 to 8 years. The lower age limit was chosen because it was felt that many children under the age of 4 years might not be registered with a dentist. The upper age limit was chosen as 8 years to assess the attitudes and experiences prior to the first orthodontic intervention of maxillary expansion, which is undertaken in many children with CLP prior to bone grafting at 8 to 9 years.

## RESULTS

Out of the hundred patients, 34% had first registered with a local dental practitioner for their cleft deformity and were then referred to the cleft center. 66% of the patients registered directly at the cleft center for their deformity. Only 20% of these children visited a local dental practitioner for their dental health care.

On evaluation of the previous dental visits, 42% had not undergone any dental treatment and received any dietary or oral hygiene advices. While, 38% of children had received dietary advice as well as general oral hygiene measures, 16% given tooth brushing instructions and 4% of the children who experienced restorative interventions (Table 1).

On assessing parental awareness regarding the treatment needs of these children, 20% of the parents were not willing to receive dietary advice or oral hygiene measures. 16% of the parents were aware regarding the various treatment modalities available to these children. 6% of the parents were apprehensive regarding the surgical correction of defects and another 30% of the parents were apprehensive about the speech problem of the child in the future. 28% of the parents knew that the speech problems could be corrected by speech therapy (Table 2).

**Table 1:** **Table 1:** Previous dental visit and patient response in the present study

***Previous dental visits***		***Patient response (%)***	
No advice on diet oral hygiene		42	
Received dietary advice and general			
oral hygiene measures		38	
Tooth brushing instructions		16	
Restoration		4	

**Table 2:** **Table 2:** Parental awareness regarding the various treatment needs

***Treatment needs***		***Patient response (%)***	
Not dietary advice		20%	
Various treatment modalities		16%	
Surgical correction of the defects		6%	
Speech problems		30%	
Speech therapy		28%	

**Graph 1:**
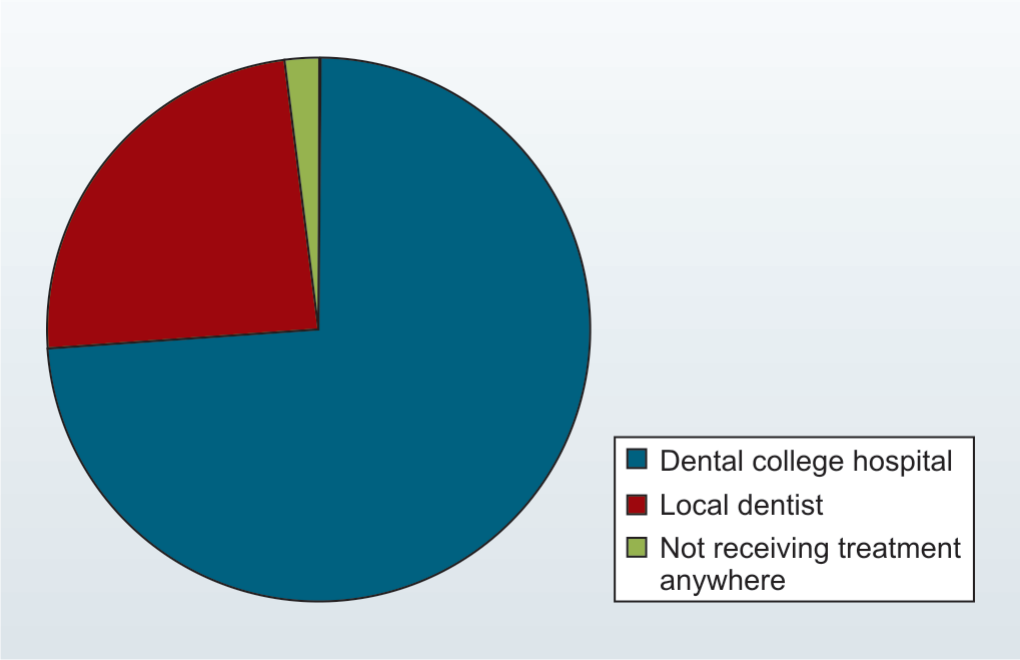
Provision of dental treatment

Regarding the provision of the dental treatment, 74% wanted a pediatric dentist to provide dental check-up and treatment at the cleft center, whereas, 24% preferred a dental practitioner close to their home and 2% does not want treatment anywhere (Graph 1).

## DISCUSSION

The provision of good pediatric dental care for children with cleft lip and palate is essential for this group of vulnerable patients. Although, coordination of care has been proposed by the *cleft team,* this is dependent on patients being able to access dental services in their locality. The present survey found that 34% of the children were registered with a dental practitioner and 66% of the children directly with the cleft center which does not follow the Clinical Standards Advising Group 1998, where 91% of children registered with a dental practitioner.^6^

In our survey, the parents of children with CLP were not much aware of the treatment needs of children, with only 16% of parents who knew the various dental treatment modalities that could be availed. Only 16% of children had received tooth brushing instruction which is very less compared to the study done by S Mc Donagh.^11^ 38% of children received dietary advice as well as general oral hygiene measures which again was found to be very less when compared to the study done by S Mc Donagh.^11^ From this survey it was found that a sizeable proportion (61%) of parents of children with CLP had not received any dental health advice.

Regarding the provision of dental examination and treatment 74% wanted the pediatric dentist to have a dental examination and treatment at the cleft center, whereas 24% preferred by a local dental practitioner. This was in agreement with the survey done by S Mc Donagh et al.^11^

The inclusion of pediatric dental support in the cleft team to identify ’at risk’ patients and facilitate the provision of care services through hospitals, community, and a general dental practitioner has been shown to be effective. This survey indicates that, there is parental support for the provision of pediatric dental assessment at the cleft center, though they prefer subsequent care with their local dental practitioners. As a pediatric dentist, we can provide anticipatory guidance to parents of these children at dental home in the cleft center. Thus, the pediatric dentist play a pivotal role as a part of the cleft team in preventing and managing dental disease and malocclusion and laying the foundation of overall health and nutrition for CLP children.

## CONCLUSION

Most parents of children with CLP prefer dental examination and preventive advices provided at the cleft center. They majorly wanted a pediatric dentist to provide the dental check-up and treatment at the cleft center.
